# Relugolix’s impact on endometriosis-associated pain and quality of life: a meta-analysis of EHP-30 outcomes

**DOI:** 10.3389/fendo.2025.1650579

**Published:** 2025-09-29

**Authors:** Jiani Xie, Xiaorong Ni, Qunhuan Huang, Ying Guo

**Affiliations:** Department of Gynecology, Shanghai Municipal Hospital of Traditional Chinese Medicine, Shanghai University of Traditional Chinese Medicine, Shanghai, China

**Keywords:** endometriosis, relugolix, GnRH antagonist, quality of life, EHP-30, pain, systematic review, meta-analysis

## Abstract

**Background:**

Relugolix offers a promising alternative for endometriosis-associated pain, yet its comprehensive impact on health-related quality of life (HRQoL), particularly as measured by the disease-specific EHP-30 questionnaire, remains underexplored.

**Methods:**

We conducted a systematic review and meta-analysis of randomized controlled trials (RCTs) investigating relugolix for endometriosis-associated pain, with a primary focus on HRQoL assessed by the Endometriosis Health Profile-30 (EHP-30). Data extracted included EHP-30 domain scores changes and proportion of EHP-30 Pain Domain Responders, along with intervention details, control type, and follow-up duration.

**Results:**

Five RCTs were included. Overall, relugolix significantly improved EHP-30 Pain domain scores (MD = 6.77, 95% CI: 3.15 to 10.39, p=0.0002) but showed substantial heterogeneity (I²=90.7%). Subgroup analysis by control type showed significant differences (p<0.0001): Relugolix was highly effective against placebo (MD = 15.31, 95% CI: 12.18 to 18.45) and placebo-matching combination therapy (MD = 8.86, 95% CI: 5.03 to 12.69), but numerically less effective than leuprorelin (MD = -3.79, 95% CI: -6.27 to -1.31). Relugolix significantly increased EHP-30 Pain Domain Responders (OR = 3.245, 95% CI: 2.496; 4.219, p < 0.0001). For other EHP-30 domains, relugolix demonstrated significant improvements in Emotional Well-being (MD = 5.71, 95% CI: 1.87; 9.55, p=0.0036), Social Support (MD = 6.40, 95% CI: 0.88; 11.93, p=0.0231), and Self-image (MD = 6.00, 95% CI: 1.03; 10.96, p=0.0179) compared to placebo.

**Conclusion:**

Oral relugolix significantly improves EHP-30 pain domain scores and patient response rates in endometriosis, particularly when compared to placebo. It also positively impacts emotional well-being, social support, and self-image.

## Introduction

Endometriosis, a chronic gynecological condition characterized by the presence of endometrial-like tissue outside the uterus, affects millions of women worldwide, leading to debilitating pain, infertility, and reduced quality of life (1). Current medical management often involves hormonal therapies, including gonadotropin-releasing hormone (GnRH) agonists and antagonists, as well as surgical interventions. While GnRH agonists have long been used, their utility is limited by hypoestrogenic side effects, such as bone mineral density (BMD) loss and vasomotor symptoms, necessitating add-back therapy for prolonged use (2, 3). Add-back therapy is the strategic use of low-dose estrogen and progestin hormones alongside GnRH antagonists to mitigate hypoestrogenic side effects, such as bone loss, without compromising the drug’s therapeutic efficacy in pain reduction.

Oral GnRH antagonists, including elagolix, relugolix, and linzagolix, represent a newer class of drugs offering oral administration and dose-dependent estrogen suppression without an initial flare-up of symptoms. This is a key advantage over GnRH agonists, which initially stimulate the pituitary, leading to a temporary surge in luteinizing hormone and follicle-stimulating hormone. This surge, known as a “flare-up,” can acutely worsen endometriosis-associated pain symptoms, including dysmenorrhea, non-menstrual pelvic pain, and dyspareunia, before their therapeutic effects take hold. The direct blockade mechanism of antagonists avoids this symptom exacerbation, offering a more immediate and tolerable pain relief pathway. Previous systematic reviews and network meta-analyses have evaluated the efficacy and safety of these oral GnRH antagonists in managing moderate-to-severe endometriosis-associated pain (EAP). For instance, Xin et al., 2022 concluded that oral GnRH antagonists were effective in reducing pelvic pain, dysmenorrhea, and dyspareunia within 12 weeks, noting dose-dependent efficacy and safety outcomes, with relugolix 40 mg being best for reducing analgesic use and having higher rates of hot flush (4). Similarly, Yan et al., 2022 found that oral GnRH antagonists were effective for EAP, dysmenorrhea, and overall patient impression, observing a dose-response relationship for ovarian hypoestrogenic effects, especially at higher doses (5). These meta-analyses have largely focused on pain reduction and common adverse events. Common adverse events associated with these therapies include hot flushes, fatigue, and headaches, which are often linked to the hypoestrogenic state induced by the drugs and may confound the interpretation of quality of life outcomes.

However, a critical gap in the existing literature is a comprehensive, focused analysis on the impact of these treatments on HRQoL, specifically as measured by the EHP-30 questionnaire. The EHP-30 is a disease-specific, patient-reported outcome measure widely recognized for its clinical value in assessing the multifaceted impact of endometriosis across various domains, including pain, control and powerlessness, social support, emotional well-being, and self-image (6). For example, the Control and Powerlessness domain assesses feelings such as being powerless and out of control, while the Emotional Well-being domain includes questions on feelings of depression or anxiety due to the condition. While some prior meta-analyses, such as that by Bafort et al., 2020 on laparoscopic surgery for endometriosis, briefly touch upon quality of life measures like the EuroQol-5D (a standardized, generic measure of health status) or the SF-12 (a widely used short-form health survey), they do not specifically focus on the EHP-30 or oral GnRH antagonists (7). EHP-30 is the preferred tool for endometriosis research as it is a disease-specific questionnaire, designed to capture the unique and multifaceted aspects of the condition that generic tools like EuroQol-5D or SF-12 may not fully address. Nogueira Neto et al., 2023 demonstrated the value of EHP-30 in assessing HRQoL improvements post-surgical treatment for endometriosis, reinforcing its utility as a key outcome measure. Despite its recognized importance, the comprehensive effects of oral GnRH antagonists on the various domains of the EHP-30 have not been the primary focus of prior meta-analyses.

Therefore, this systematic review and meta-analysis aims to specifically synthesize and critically evaluate the evidence regarding the efficacy and safety of oral GnRH antagonists, with a particular emphasis on their impact on HRQoL as assessed by the EHP-30 questionnaire. By concentrating on this clinically valuable and specific HRQoL measure, this study seeks to provide a more nuanced understanding of the patient-centered benefits of these novel therapies beyond pain relief alone.

## Methods

This systematic review and meta-analysis was conducted in accordance with the Preferred Reporting Items for Systematic Reviews and Meta-Analyses (PRISMA) guidelines (8) and generally followed the methodological recommendations outlined in the Cochrane Handbook for Systematic Reviews of Interventions.

### Search strategy

A comprehensive systematic search was performed across major electronic databases, including PubMed, Embase, Cochrane Library, and Web of Science, to identify relevant randomized controlled trials. The search strategy incorporated a combination of Medical Subject Headings (MeSH) terms and free-text keywords related to relugolix, GnRH antagonists, endometriosis, and quality of life. The search terms included, but were not limited to: (“relugolix” OR “Myfembree” OR “Ryeqo” OR “GnRH antagonist*” OR “gonadotropin-releasing hormone antagonist*”) AND (“endometriosis” OR “endometriosis-associated pain” OR “dysmenorrhea” OR “pelvic pain”) AND (“EHP-30” OR “Endometriosis Health Profile-30” OR “quality of life”) AND (“randomized controlled trial*” OR “RCT*” OR “clinical trial*”). Specific MeSH terms such as “Endometriosis”[Mesh], “Gonadotropin-Releasing Hormone Antagonists”[Mesh], “Relugolix”[Mesh], “Quality of Life”[Mesh], “Pain”[Mesh], and “Randomized Controlled Trials as Topic”[Mesh] were used where applicable to maximize search sensitivity. The search was updated up to the date of analysis.

#### Inclusion criteria

Randomized controlled trials (RCTs) that investigated relugolix (as monotherapy or combination therapy) for the treatment of EAP.

Studies that reported outcomes related to health-related quality of life, specifically utilizing the EHP-30 questionnaire.

Studies involving pre-menopausal women diagnosed with endometriosis.

#### Exclusion criteria

Non-randomized studies, such as cohort studies, case reports, observational studies, reviews, and meta-analyses.

*In vitro* or animal studies.

Studies that did not include relugolix as an intervention.

Studies that did not report EHP-30 outcomes.

Studies focusing solely on other GnRH antagonists without a relugolix arm.

### Data extraction

Two independent reviewers meticulously screened titles and abstracts, followed by full-text review of potentially eligible articles. Any discrepancies between the reviewers were resolved through discussion or, if necessary, by a third independent reviewer. For each eligible study included in the meta-analysis, comprehensive data pertaining to the control arms were systematically extracted. This involved identifying the specific type of comparator employed, which broadly fell into categories such as pure placebo, active comparator (e.g., another established therapeutic agent like leuprorelin), or more complex placebo designs, such as those used to maintain blinding in combination therapy studies (e.g., placebos designed to match active combination therapy components). For each identified control group, critical quantitative data were extracted, including the number of participants randomized to that group, the mean baseline EHP-30 pain domain score, the mean change from baseline in EHP-30 pain domain score, and the corresponding standard deviation for both the baseline and change scores. Information regarding the study’s blinding strategy, particularly in cases involving double-dummy designs or active placebo regimens, was also noted to understand the nature of the control intervention. This systematic extraction facilitated subsequent subgroup analyses based on the nature of the control arm, enabling a nuanced examination of the relative efficacy of the intervention under different comparative conditions.

Additional extracted data included: author and year of publication, country of study, study period, study design (phase, blinding, control group type), total sample size, participant demographics, specific intervention drugs (relugolix dosage, components of combination therapy), primary and secondary endpoints, and key exclusion criteria. For relevant studies, clinical trial registration numbers were also recorded.

### Risk of bias assessment

**Figure 1:**
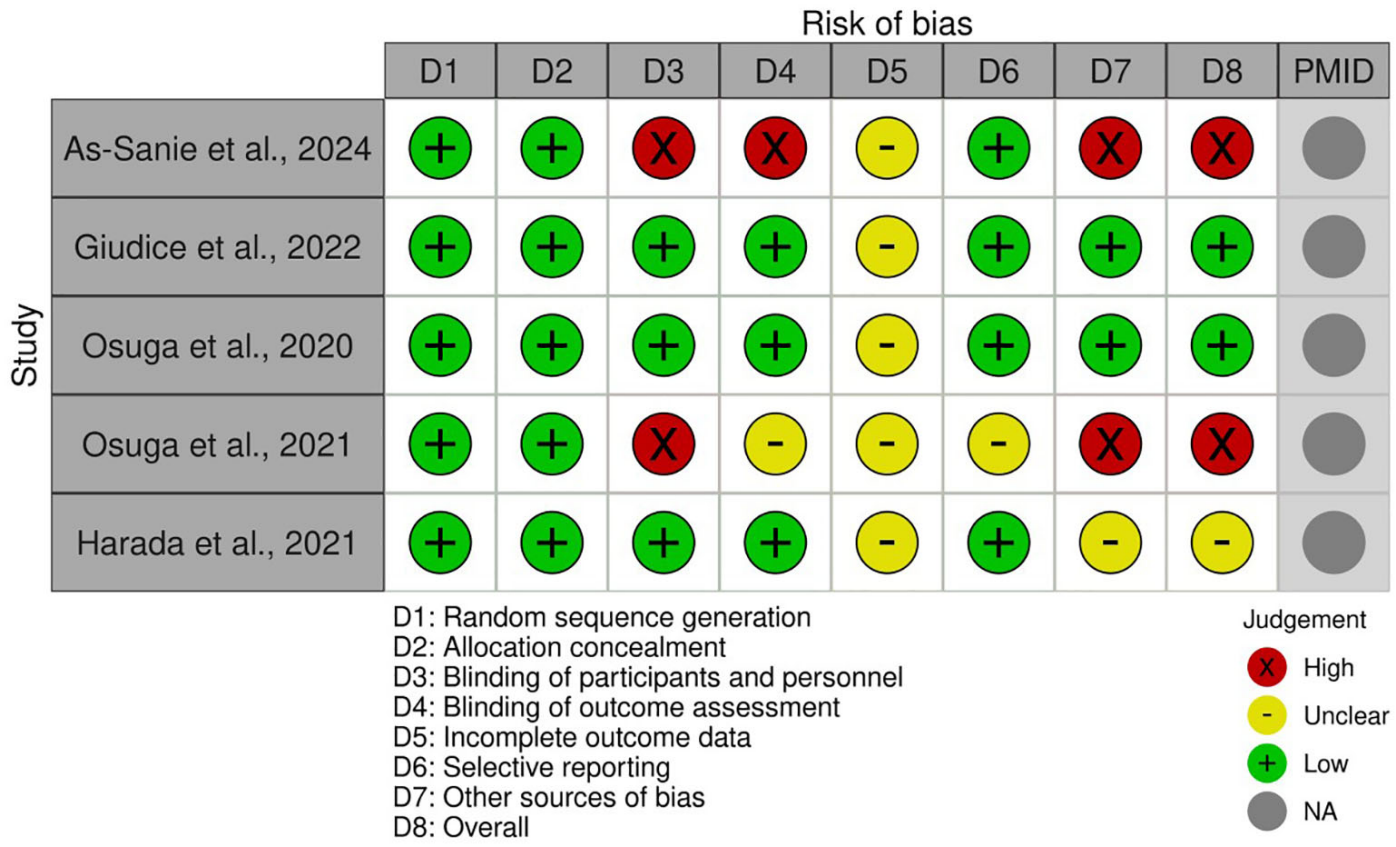
Plot of the risk of bias assessment for each included studies.

The risk of bias for each included RCT was assessed independently by two reviewers using a modified version of SYRCLE’s risk of bias tool (9). This tool evaluates studies across eight domains: allocation concealment, random sequence generation, blinding of personnel and participants, selective reporting, blinding of outcome assessment, incomplete outcome data, and other sources of bias. Each domain was judged as having a “low,” “high,” or “unclear” risk of bias. If there were disagreements between reviewers, they would be resolved by reaching consensus or by consulting a third reviewer. The overall risk of bias for each study was determined based on the assessment of these individual domains. The results of the risk of bias assessment are visualized in **Figure 1**.

### Statistical analyses

All statistical analyses were performed using R statistical software (10). For continuous outcomes, such as mean changes in EHP-30 domain scores, the Mean Difference (MD), which represents the average difference in outcome between the treatment and control groups, and its 95% confidence interval (CI) were calculated. For dichotomous outcomes, such as the proportion of EHP-30 Pain Domain Responders, the Odds Ratio (OR) and its 95% CI were calculated. Heterogeneity between studies was assessed using Cochrane’s Q test and the I² statistic. An I² value of <25% was considered to indicate low heterogeneity, 25-50% moderate heterogeneity, and >50% substantial heterogeneity. Due to the anticipated clinical and methodological heterogeneity across the included studies (e.g., variations in study design, intervention dosages, and follow-up durations), a random-effects model (e.g., DerSimonian-Laird method or restricted maximum likelihood) was employed for all meta-analyses to account for both between-study and within-study variance. Q-statistic for subgroup differences was calculated to formally assess whether there were statistically significant differences in effect sizes across subgroups. p-value < 0.05 was considered statistically significant.

## Results

### Study selection process

**Figure 2:**
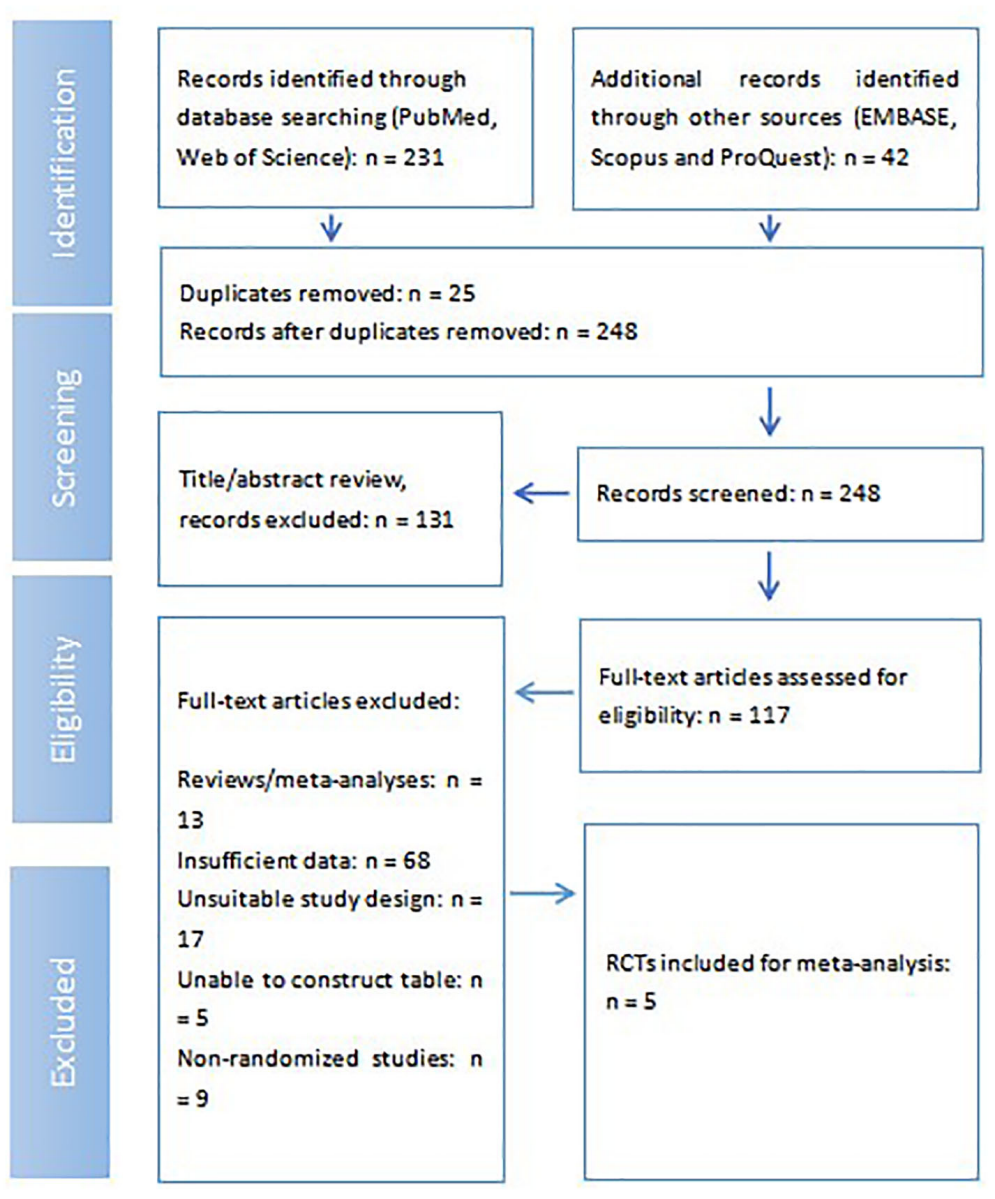
Flow diagram of study selection process.

The study selection process, shown as **Figure 2**, adhered to systematic review guidelines. An initial comprehensive search across PubMed and Web of Science yielded 231 records, complemented by an additional 42 records identified from other sources, specifically EMBASE, Scopus, and ProQuest. After removing 25 duplicate entries, a total of 248 unique records proceeded to the screening phase. During the title and abstract review, 131 records were deemed irrelevant and excluded, leaving 117 full-text articles for detailed eligibility assessment. Following a thorough full-text review, 112 articles were excluded for various reasons: 13 were reviews or meta-analyses, 68 had insufficient data for extraction, 17 featured an unsuitable study design, 5 were unable to have their data tabulated, and 9 were identified as non-randomized studies. Consequently, five RCTs met all predefined inclusion criteria and were incorporated into the final meta-analysis (11–15) (**Table 1**).

**Table 1:** Characteristics of Included RCTs.

Study	Country	Study period	Study design	Intervention drugs	Primary endpoint	Secondary endpoint	Exclusion criteria
As-Sanie et al., 2024 (11)	Multiple countries across Africa, Australasia, Europe, North America, and South America	Up to 104 weeks (2 years) for this analysis.	Long-term extension (LTE) study of preceding randomized SPIRIT studies. It is an open-label, prospective cohort study where participants continue treatment based on their original randomization. RCT registration numbers are not provided in this specific text, but it references “randomized SPIRIT studies” and “SPIRIT LTE study” suggesting they would be found in those references.	Relugolix CT (Combination Therapy): All participants in the LTE received a co-packaged relugolix (40 mg) tablet and estradiol (1 mg) as well as norethindrone acetate (0.5 mg) capsule once daily for 80 weeks. Note: While all LTE participants received relugolix CT, analyses were performed based on original randomization groups from the pivotal studies: relugolix CT, delayed relugolix CT (relugolix 40 mg alone for 12 weeks, then relugolix CT for 12 weeks), or placebo. This study itself did not involve new intervention groups or control arms within the LTE.	The EHP-30 pain domain was a predefined secondary endpoint of the SPIRIT LTE study. The primary focus of this specific analysis is to assess the effect of relugolix CT on functioning and QoL for up to 104 weeks based on the EHP-30 questionnaire.	Non-Pain domains (Social Support, Control and Powerlessness, Emotional Well-Being, Self-Image) and the EHP-30 total score were exploratory endpoints. Safety outcomes were also assessed. A *post hoc* analysis assessed the relationship between changes in dysmenorrhea and NMPP NRS scores and changes in EHP-30 domains and total scores.	Women with a Z-score < -2.0 or a ≥7% decrease in BMD from the pivotal study baseline at the total hip, lumbar spine, or femoral neck were excluded from this LTE.
Giudice et al., 2022 (12)	Multiple countries across Africa, Australasia, Europe, North America, and South America	The treatment period was 24 weeks with a subsequent 30-day safety follow-up.	Two replicate, phase 3, multicenter, randomized, double-blind, placebo-controlled studies. RCT Registration Numbers: SPIRIT 1: ClinicalTrials.gov (NCT03204318) and EudraCT (2017–001588–19); SPIRIT 2: ClinicalTrials.gov (NCT03204331) and EudraCT (2017–001632–19).	Relugolix Combination Therapy: Once-daily relugolix 40 mg orally as a tablet and estradiol 1 mg and norethisterone acetate 0.5 mg orally as a capsule. Delayed Relugolix Combination Therapy: Relugolix 40 mg monotherapy for 12 weeks, followed by relugolix combination therapy (relugolix 40 mg + estradiol 1 mg + norethisterone acetate 0.5 mg) for 12 weeks. Placebo: Placebo tablet and placebo capsule, manufactured to match the active treatments in size, shape, and color.	The proportion of responders at Week 24 based on the dysmenorrhea NRS score, and the proportion of responders at Week 24 based on the non-menstrual pelvic pain NRS score, both comparing the relugolix combination therapy group with the placebo group.	The proportion of responders at Week 24 based on the dysmenorrhea NRS score, and the proportion of responders at Week 24 based on the non-menstrual pelvic pain NRS score, both comparing the relugolix combination therapy group with the placebo group.	Patients with a bone mineral density Z score of less than –2.0 at specific sites, history of chronic pelvic pain not caused by endometriosis, or a contraindication to combined hormonal therapy.
Osuga et al., 2020 (13)	Japan	December 2011 and September 2013 (treatment period 12 weeks, with a 12-week extension possibility for a total of 24 weeks)	Phase 2, multicenter, randomized, double-blind, parallel-group, placebo-controlled study. Clinical Trial Registration Number: NCT01458301	Experimental Groups: Relugolix 10 mg, 20 mg, or 40 mg once daily oral tablets, with leuprorelin-placebo subcutaneous injections; Control Groups: Placebo oral tablets with leuprorelin-placebo subcutaneous injections; or Leuprorelin 3.75 mg subcutaneous injections every 4 weeks with relugolix-placebo oral tablets.	Change from baseline in mean VAS score for pelvic pain during the 28 days before the end of the treatment period.	Change from baseline in VAS scores for pelvic pain and dyspareunia during the treatment period; treatment-emergent adverse events (TEAEs); bone mineral density (BMD); vital signs; body weight changes; 12-lead electrocardiogram; clinical laboratory test results; modified Biberoglu and Behrman (M-B&B) score; Biberoglu and Behrman (B&B) score; analgesic use; Endometriosis Health Profile-30 (EHP-30) score; serum concentrations of estradiol, luteinizing hormone, follicle-stimulating hormone, and progesterone.	Patients with measurable uterine fibroids (longest diameter ≥3 cm), lower abdominal pain from irritable bowel syndrome or severe interstitial cystitis, thyroid dysfunction, pelvic inflammatory disease, positive Papanicolaou smear, history of hysterectomy or bilateral oophorectomy, or serious cardiovascular, hepatic, renal, or hematologic disorders.
Osuga et al., 2021 (14)	Japan	March 2012 and February 2014 (overall treatment duration was 24 weeks, including the preceding study’s 12 weeks plus this extension’s additional 12 weeks)	Phase 2, multicenter, long-term extension study (Open-label extension of a preceding RCT). RCT registration number: NCT01452685	Experimental Groups: Relugolix 10 mg, 20 mg, or 40 mg daily oral tablets (continued from preceding study); Control Groups: Placebo oral tablets (continued from preceding study); or Leuprorelin 3.75 mg subcutaneous injections every 4 weeks (continued from preceding study).	Assessments of safety, including bone mineral density (BMD), treatment-emergent adverse events (TEAEs), vital signs, weight, 12-lead electrocardiogram (ECG), and clinical laboratory tests.	Visual analog scale (VAS) scores for pelvic pain, dysmenorrhea, and dyspareunia; modified Biberoglu and Behrman (M-B&B) and B&B scales for EAP symptoms; analgesic use; decrease in menstrual blood loss; achievement of amenorrheic state; quality of life assessed by Endometriosis Health Profile-30 (EHP-30); and blood concentration of E2, P, LH, and FSH.	Patients were excluded if they experienced treatment-emergent adverse events in the preceding study that made continuation unsafe, were unable to comply with the protocol due to new/aggravated conditions, showed no efficacy in the preceding study, or developed symptoms of hypoestrogenism.
Harada et al., 2021 (15)	Japan	May 2019 to June 2020 (treatment period 24 weeks with a 4-week follow-up)	Phase 3, multicenter, randomized, double-blind, double-dummy, active-controlled study. Clinical Trial Registration Number: NCT03931915	Experimental Group (REL): Relugolix (REL) 40 mg daily oral tablet; Control Group (LEU): Leuprorelin (LEU) 3.75 mg or 1.88 mg subcutaneous injection every 4 weeks (1.88 mg for patients with body weight <50 kg), administered in a double-dummy design with corresponding placebo oral tablets.	Change in the maximum Visual Analog Scale (VAS) score from baseline to the end of the treatment period (EOT) for endometriosis-associated pelvic pain.	Change from baseline to EOT in mean VAS score, menstrual pain, non-menstrual pelvic pain (NMPP), dyspareunia, Biberoglu and Behrman (B&B) score, Endometriosis Health Profile-30 (EHP-30) score, and Work Productivity and Activity Impairment Questionnaire: General Health (WPAI-GH) score.	Patients were excluded if they used certain medications (e.g., bisphosphonates, GnRH analogs, sex hormones), had specific medical histories (e.g., hysterectomy, bilateral oophorectomy, uterine fibroid requiring treatment), suffered from other confounding pain conditions (e.g., IBS), or had serious cardiovascular, hepatic, renal, or hematologic disorders.

### Characteristics of included RCTs

The included RCTs primarily investigated relugolix and its combination therapies for EAP and quality of life. Geographically, these studies spanned multinational sites (11, 12) and Japan (13–15). The study designs varied by phase and duration; initial pivotal trials (12, 13, 15) were typically 12 to 24-week, multicenter, randomized, double-blind, and placebo or active-controlled, while long-term extension studies (11, 14) followed an open-label, prospective cohort design, extending treatment up to 104 weeks. Intervention arms consistently featured relugolix (in various doses or as a 40 mg combination therapy with estradiol and norethisterone acetate) compared against placebo or leuprorelin. Primary endpoints largely focused on changes in dysmenorrhea and non-menstrual pelvic pain measured by Numerical Rating Scale (NRS) or Visual Analog Scale (VAS) scores, with secondary endpoints often encompassing broader quality of life assessments (EHP-30), dyspareunia, bone mineral density, and safety profiles. Common exclusion criteria included pre-existing conditions that could confound pain assessment, contraindications to hormonal therapies, or significant comorbidities, aiming to ensure a relatively homogenous study population (**Table 1**).

### Risk of bias assessment

It presents the risk of bias assessment for each included study across eight domains, utilizing a modified SYRCLE’s risk of bias tool shown as **Figure 1**. Across the studies, “Random sequence generation” and “Allocation concealment” consistently demonstrated a low risk of bias, indicating robust randomization procedures. However, variability was observed in other domains. “Blinding of participants and personnel” and “Blinding of outcome assessment” showed a higher risk of bias, particularly in extension or open-label studies (11, 14). “Incomplete outcome data” and “Selective reporting” often presented an unclear risk, highlighting a lack of detailed reporting on missing data handling or protocol pre-registration. “Other sources of bias” varied, with some studies demonstrating low risk while others, notably extension studies, presented a high risk due to factors such as selective patient enrollment or changes in study design.

### Stratified meta-analysis of relugolix’s effect by comparator arm

**Figure 3:**
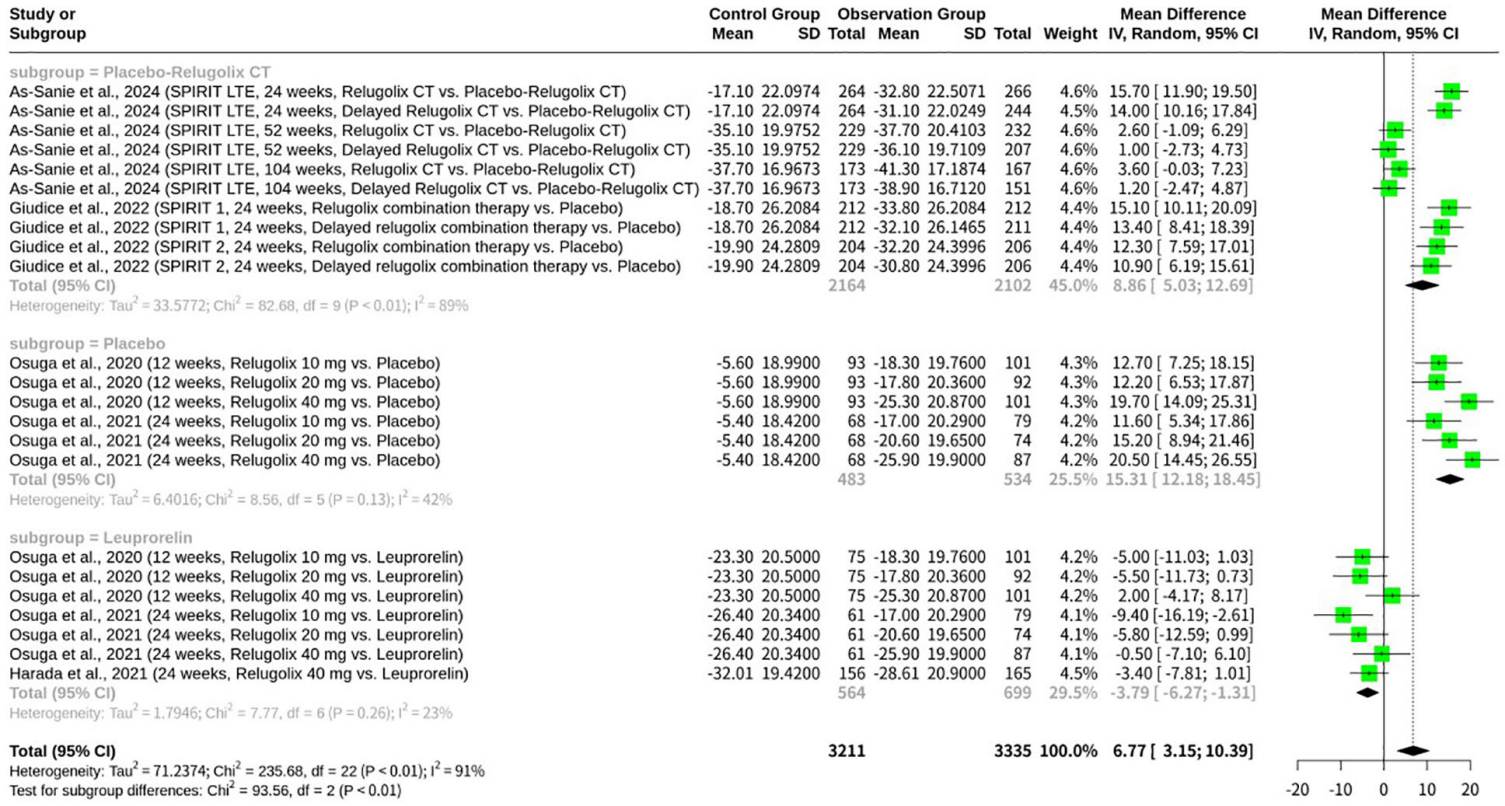
Forest plot of mean difference in EHP-30 pain domain scores, subgrouped by control type.

The meta-analysis, shown as **Figure 3**, investigating the clinical efficacy and safety of Relugolix for EAP, specifically examined the EHP-30 pain domain scores. The overall random effects model (REM) revealed a MD of 6.77 (95% CI: 3.15 to 10.39, z=3.66, p=0.0002), indicating a significant effect. However, substantial heterogeneity was present across all studies (I^2^ = 90.7%, Q = 235.68, df=22, p<0.0001). A key finding from the subgroup analysis, which was stratified by the type of control group, showed significant differences between these subgroups (Q = 93.56, df=2, p<0.0001). Specifically, studies with a “Placebo-Relugolix CT” control group yielded an MD of 8.86 (95% CI: 5.03 to 12.69) with high within-subgroup heterogeneity (I2 = 89.1%). Studies utilizing a “Placebo” control group demonstrated a larger MD of 15.31 (95% CI: 12.18 to 18.45) with moderate heterogeneity (I^2^ = 41.6%). Conversely, studies with “Leuprorelin” as the active control showed an MD of -3.79 (95% CI: -6.27 to -1.31) with low heterogeneity (I^2^ = 22.8%). This negative mean difference suggests that Relugolix, when compared to Leuprorelin, resulted in a numerically smaller improvement (or a slightly worse outcome) in EHP-30 pain domain scores.

### Stratified meta-analysis of relugolix’s effect by dosage of relugolix

**Figure 4:**
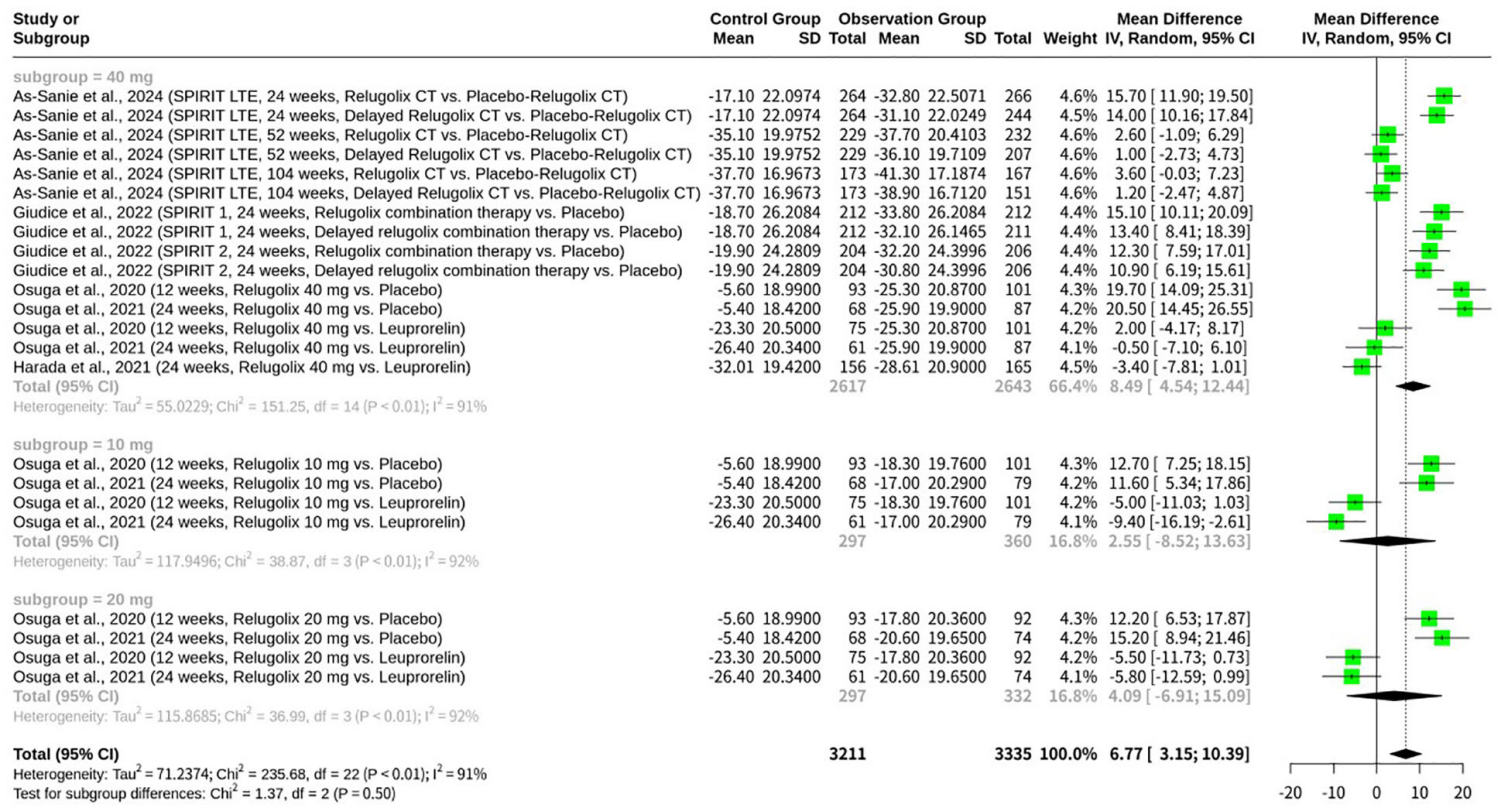
Forest plot of mean difference in EHP-30 pain domain scores, subgrouped by dosage of relugolix.

Subgroup analysis is also conducted based on the dosage of Relugolix administered in the experimental group. The meta-analysis, shown as **Figure 4**, reveals a significant overall MD in favor of Relugolix, indicating its efficacy in improving EHP-30 pain domain scores. However, substantial heterogeneity was observed across studies (I^2^ = 90.7%), suggesting considerable variability in treatment effects. Subgroup analysis by Relugolix dosage showed a greater effect in the 40 mg subgroup (MD = 8.49; 95%-CI: 4.54; 12.44; I^2^ = 90.7%), compared to the 10 mg (MD = 2.55; 95%-CI: -8.52; 13.63; I^2^ = 92.3%) and 20 mg (MD = 4.09; 95%-CI: -6.91; 15.09; I^2^ = 91.9%) subgroups. Despite the observed differences in subgroup mean effects, the test for subgroup differences did not reach statistical significance (Q = 1.37; d.f. = 2; p = 0.5031), indicating that the effect of Relugolix on EHP-30 pain domain scores does not significantly differ across the 10 mg, 20 mg, and 40 mg dosage subgroups.

### Stratified meta-analysis of relugolix’s effect by relugolix administration as monotherapy versus combination therapy

**Figure 5:**
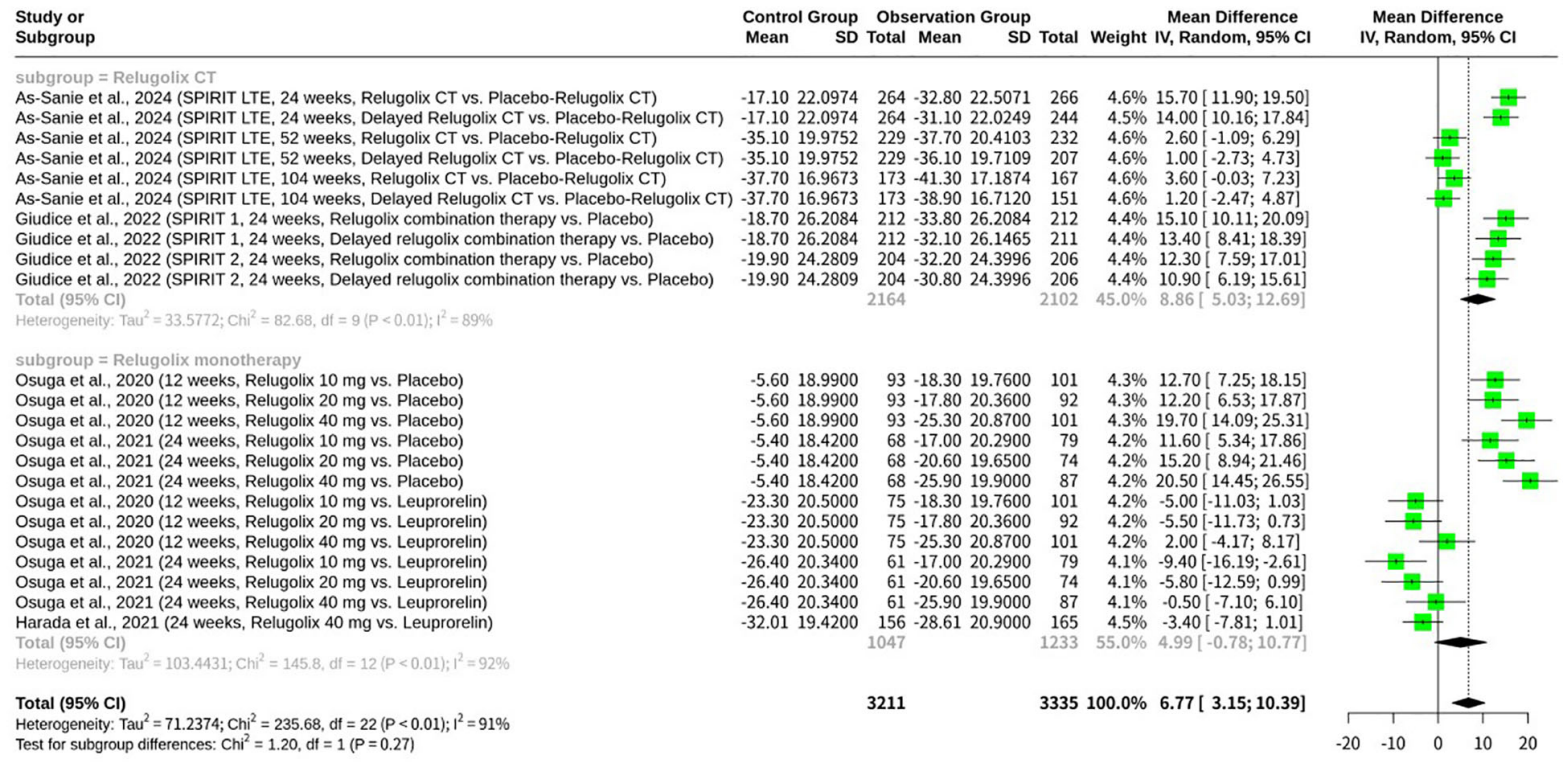
Forest plot of mean difference in EHP-30 pain domain scores, subgrouped by relugolix administration as monotherapy versus combination therapy.

This meta-analysis, shown as **Figure 5**, also conducted a stratified analysis to investigate the impact of Relugolix administration as monotherapy versus combination therapy (Relugolix CT) on EHP-30 pain domain scores. When analyzing the subgroups, Relugolix combination therapy demonstrated a mean difference of 8.86 (95%-CI: 5.03; 12.69) across 10 observations, suggesting a robust effect in pain reduction, albeit with substantial internal heterogeneity (I^2^ = 89.1%). Similarly, Relugolix monotherapy, based on 13 observations, showed a mean difference of 4.99 (95%-CI: -0.78; 10.77). While the confidence interval for monotherapy crossed zero, indicating a less conclusive effect on its own, it still trended towards benefit. Despite the numerical difference in mean effects between the combination therapy and monotherapy subgroups, the formal test for subgroup differences did not yield statistical significance (Q = 1.20, d.f. = 1, p=0.2740). This finding suggests that, based on the current evidence, there is no significant difference in the magnitude of pain reduction achieved with Relugolix when administered as part of a combination therapy versus as a monotherapy.

### Stratified meta-analysis of relugolix’s effect by follow-up duration

**Figure 6:**
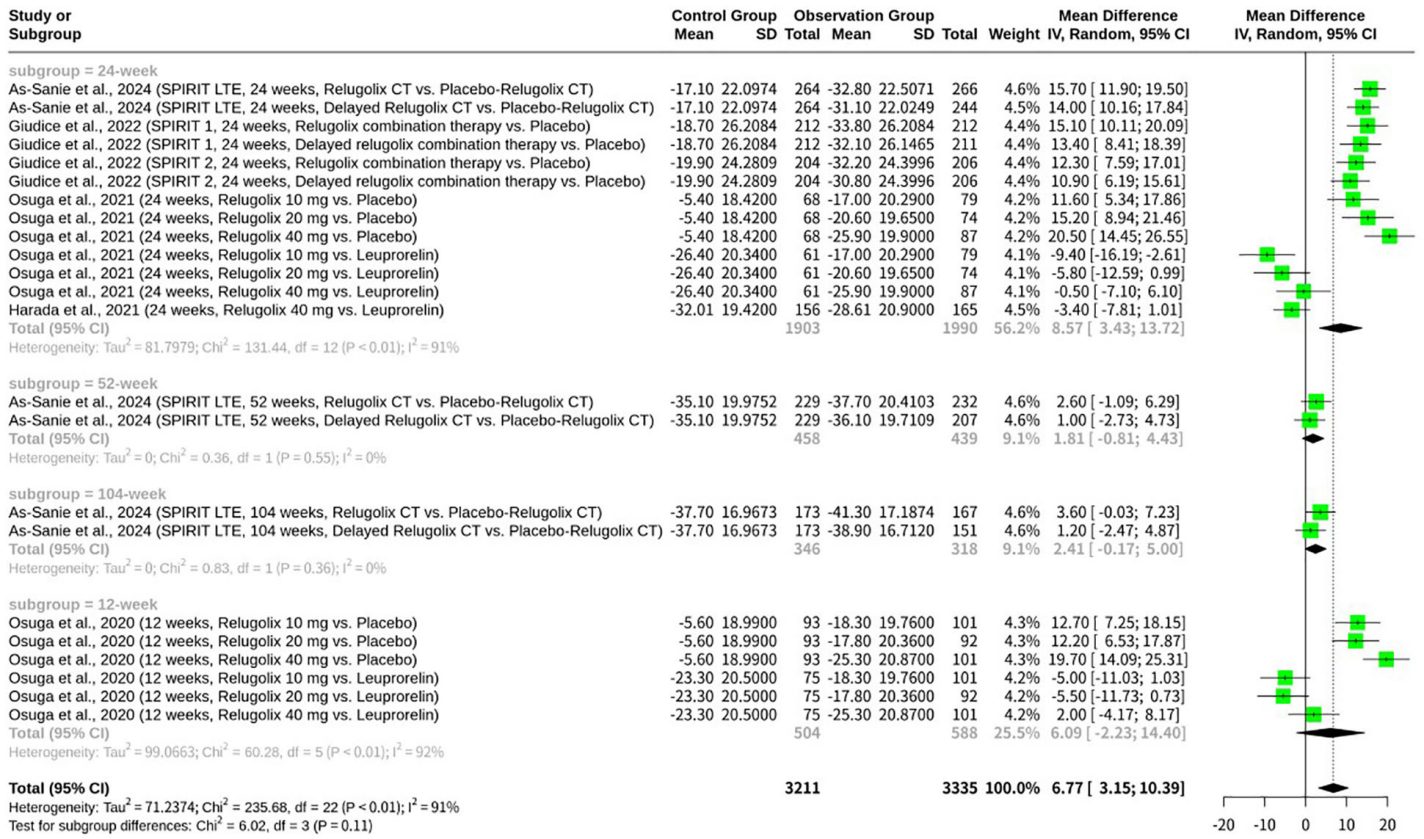
Forest plot of mean difference in EHP-30 pain domain scores, subgrouped by follow-up duration.

Further stratified analysis, shown as **Figure 6**, was conducted to assess the impact of follow-up duration on the observed efficacy of Relugolix on EHP-30 pain domain scores. This subgrouping categorized studies into 12-week, 24-week, 52-week, and 104-week follow-up periods. Within the subgroups, the 24-week follow-up duration, encompassing 13 observations, showed the largest mean difference in pain reduction (MD = 8.57; 95%-CI: 3.43; 13.72), although it also retained high internal heterogeneity (I^2^ = 90.9%). The 12-week subgroup also indicated a positive mean difference (MD = 6.09; 95%-CI: -2.23; 14.40), but its confidence interval crossed zero, making its standalone effect less definitive, and it too exhibited high heterogeneity (I^2^ = 91.7%). In contrast, the longer-term follow-up subgroups (52-week and 104-week), each comprising only two observations, presented smaller mean differences (MD = 1.81 for 52-week; MD = 2.41 for 104-week). Notably, these longer-term subgroups showed no heterogeneity (I^2^ = 0.0%), which is likely attributable to the limited number of studies in these categories rather than a true absence of variability. Their respective confidence intervals for the mean difference both crossed zero (52-week: [-0.81; 4.43]; 104-week: [-0.17; 4.99]), indicating that a significant benefit in EHP-30 pain scores could not be conclusively demonstrated at these extended durations in the current analysis. Despite these numerical variations across follow-up periods, the formal test for subgroup differences did not reach statistical significance (Q = 6.02, d.f. = 3, p=0.1104). This suggests that the effect of Relugolix on EHP-30 pain domain scores does not significantly differ based on the duration of follow-up included in the current meta-analysis.

### Meta-analysis of relugolix’s effect on EHP-30 pain domain responders

**Figure 7:**
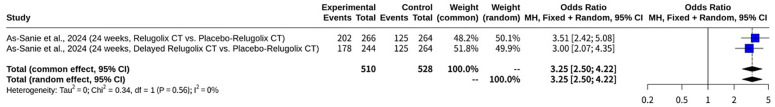
Forest plot of odds ratio of relugolix’s effect on EHP-30 pain domain responders.

The meta-analysis, shown as **Figure 7**, was conducted to assess the effect of Relugolix on EHP-30 Pain Domain Responders, combining data from two datasets by As-Sanie et al., 2024 (SPIRIT LTE, 24 weeks). A total of 1038 observations, with 630 reported responders, were included. The meta-analysis, utilizing both common and random effects models, yielded a significant overall odds ratio (OR = 3.245, 95%-CI: 2.496; 4.219, p < 0.0001). This indicates that patients receiving Relugolix were approximately 3.25 times more likely to be EHP-30 Pain Domain Responders compared to the control group. Importantly, the analysis revealed no heterogeneity across the two included studies (I^2^ = 0.0%, Q = 0.34, d.f. = 1, p=0.5571), suggesting a highly consistent effect between them.

### Assessing patient experience with EHP-30 pain scores

The EHP-30 has emerged as a particularly valuable tool in endometriosis research, as it moves beyond simple pain intensity to assess a condition’s full impact on a patient’s daily functioning and emotional well-being. A higher score on the EHP-30 indicates a greater negative impact on quality of life (11). While the As-Sanie paper provides a comprehensive discussion of EHP-30 outcomes, the findings presented here are derived from a broader meta-analysis that systematically aggregates data from multiple studies. Our analysis is not a simple repetition but a synthesis of the pooled evidence, including the As-Sanie data, to provide a more robust and statistically powered overview of the overall treatment effect. A robust body of clinical trial evidence consistently establishes relugolix, an oral GnRH antagonist, as an effective treatment for endometriosis-associated pain. These studies have shown statistically significant improvements in pain symptoms compared to placebo, with pain reduction often starting within the first month of treatment. Notably, the therapeutic effect appears to be dose-dependent, with the 40 mg dose yielding the most significant improvements (12, 14). The efficacy of relugolix extends to both menstrual and non-menstrual pelvic pain, and long-term studies like the SPIRIT trials have documented sustained pain relief over a period of up to 104 weeks. These long-term outcomes show a strong correlation between pain reduction and improvements in EHP-30 pain domain scores and overall quality of life (11). Furthermore, comparative studies, such as a Phase 3 trial conducted in Japanese women, found that relugolix was non-inferior to leuprorelin, a traditional GnRH agonist, in reducing pelvic pain. Both treatments successfully reduced VAS scores below the daily pain threshold of 30, suggesting a minimal impact on daily activities (15). This extensive body of evidence highlights that relugolix not only alleviates pain but also produces clinically meaningful benefits that tangibly improve patients’ daily lives, as comprehensively reflected by the EHP-30.

### Meta-analysis of relugolix’s effect on EHP-30 control and powerlessness domain

**Figure 8:**
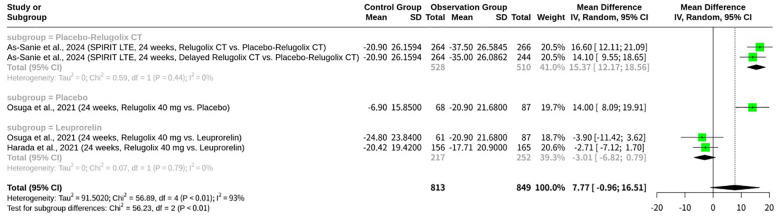
Forest plot of odds ratio of relugolix’s effect on EHP-30 control and powerlessness domain.

The meta-analysis, shown as **Figure 8**, investigated the effect of Relugolix on the EHP-30 Control and Powerlessness domain scores, synthesizing data from five datasets. The overall REM yielded a non-significant mean difference (MD = 7.77, 95%-CI: -0.96; 16.51, p=0.0811) but revealed substantial heterogeneity (I^2^ = 93.0%, p<0.0001). A subgroup analysis, stratified by the control group’s intervention, demonstrated a significant difference between subgroups (p<0.0001). Specifically, Relugolix significantly improved control and powerlessness scores when compared to placebo or delayed Relugolix combination therapy (MD = 15.37, 95%-CI: 12.17; 18.56 for “Placebo-Relugolix CT” subgroup, I^2^ = 0.0%; MD = 14.00, 95%-CI: 8.09; 19.91 for “Placebo” subgroup). However, when compared to Leuprorelin, another active GnRH agonist, Relugolix did not show a significant improvement (MD = -3.01, 95%-CI: -6.82; 0.79 for “Leuprorelin” subgroup, I^2^ = 0.0%), numerically favoring Leuprorelin.

The concepts of control and powerlessness are central to psychological well-being, defined as a person’s perceived ability to influence life events and the opposing feeling that external forces dictate outcomes, respectively (14). In clinical trials, these concepts are often measured using standardized scales like the EHP-30, where domain scores reflect patient-reported outcomes (11). A study on Relugolix’s clinical impact specifically noted changes in “Control and Powerlessness” domain scores over time, with data presented by visit up to 104 weeks for groups including a placebo (11). The data showed that a change in a domain score could be significant, for example, a score changing by 55 points from baseline (11). Without knowing the scale range, a decrease of 55 points could suggest a greater sense of powerlessness (11). However, the specific context of the EHP-30 domain suggests that a change of this magnitude would be a critical indicator of altered psychological well-being (11). This demonstrates how therapeutic interventions, such as Relugolix, can be evaluated not just on physiological outcomes but also on their effect on patients’ perceived control and agency over their health and life.

### Meta-analysis of relugolix’s effect on EHP-30 emotional well-being domain

**Figure 9:**
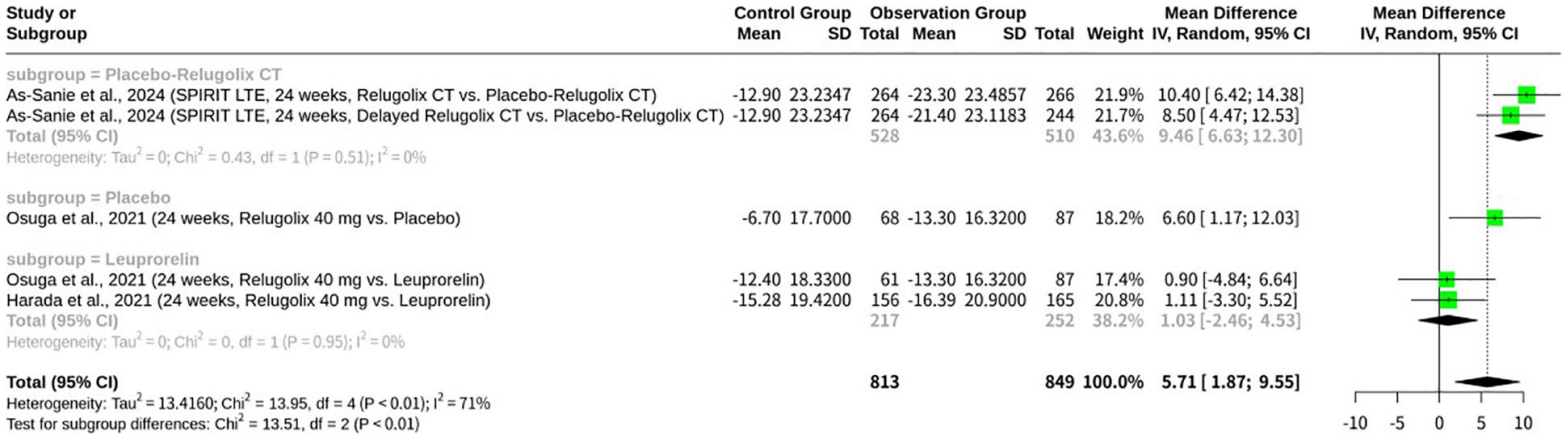
Forest plot of odds ratio of relugolix’s effect on EHP-30 emotional well-being domain.

This meta-analysis, shown as **Figure 9**, synthesized data from five datasets to evaluate the effect of Relugolix on EHP-30 Emotional Well-being domain scores. The overall REM revealed a significant mean difference of 5.71 (95%-CI: 1.87; 9.55, p=0.0036), indicating a general improvement in emotional well-being with Relugolix. However, substantial heterogeneity was observed across studies (I^2^ = 71.3%, p=0.0075). A subgroup analysis, stratified by the control group’s intervention, showed a highly significant difference between subgroups (p=0.0012). Specifically, Relugolix demonstrated a significant improvement in emotional well-being when compared to a placebo or delayed Relugolix combination therapy (MD = 9.46, 95%-CI: 6.63; 12.30 for the “Placebo-Relugolix CT” subgroup, and MD = 6.60, 95%-CI: 1.17; 12.03 for the “Placebo” subgroup), with no heterogeneity within these subgroups. In contrast, when compared against Leuprorelin, another active GnRH agonist, Relugolix’s effect on emotional well-being was not significant (MD = 1.03, 95%-CI: -2.47; 4.53), suggesting comparable efficacy between the two active treatments.

Emotional well-being is a multifaceted concept that is frequently assessed in health-related studies to gauge a patient’s overall quality of life (15) (11). In clinical research, domain scores on scales like the EHP-30 are used as patient-reported outcome measures (PROMs) to quantify changes in a patient’s psychological state during treatment (11). For example, the study might report that an “LS mean score improved by 45” in the emotional well-being domain (11). This type of data indicates a substantial positive change in participants’ psychological well-being as a result of the intervention (11). The literature also suggests that the number associated with emotional well-being, such as “21” or “23”, may refer to a specific score, a patient’s age, or a numbered item on an assessment (12) (15). These fragments underscore the significance of emotional well-being as a key metric in evaluating the full impact of a therapeutic intervention.

### Meta-analysis of relugolix’s effect on EHP-30 social support domain

**Figure 10:**
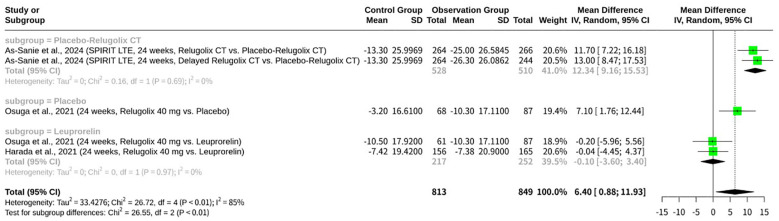
Forest plot of odds ratio of relugolix’s effect on EHP-30 social support domain.

This meta-analysis, shown as **Figure 10**, assessed the effect of Relugolix on the EHP-30 Social Support Domain scores, integrating data from five datasets. The overall REM indicated a significant positive mean difference (MD = 6.40, 95%-CI: 0.88; 11.93, p=0.0231), suggesting an improvement in social support aspects with Relugolix. However, significant heterogeneity was identified across the studies (I^2^ = 85.0%, p<0.0001). A subgroup analysis, categorized by the control group’s intervention, revealed a highly significant difference between subgroups (p<0.0001). Relugolix demonstrated a significant improvement in social support scores when compared to a placebo or delayed Relugolix combination therapy (MD = 12.34, 95%-CI: 9.16; 15.53 for the “Placebo-Relugolix CT” subgroup; MD = 7.10, 95%-CI: 1.76; 12.44 for the “Placebo” subgroup), with no heterogeneity observed within these subgroups. In contrast, when compared against Leuprorelin, another active GnRH agonist, Relugolix showed no significant effect on social support (MD = -0.10, 95%-CI: -3.60; 3.40), with the confidence interval crossing zero.

Social support is a critical factor in patient well-being, encompassing emotional, informational, and practical support from family, friends, and the community (11). In clinical trials, the social support domain is often measured using validated questionnaires to track changes over time (11). For example, one study examined changes in the social support domain over a period of 104 weeks in a placebo-controlled clinical trial for Relugolix (11). Data from such studies can show significant improvements, with scores increasing by a notable margin, such as “46 points,” indicating a stronger sense of social connectedness among participants (11). The number associated with “social support,” such as “16” or “12,” may refer to a specific scale item, a number of social support types, or a section within a questionnaire (12) (15). These findings highlight how therapeutic interventions can have a measurable positive impact on a patient’s perceived social support, an essential component of their overall health and resilience.

### Meta-analysis of relugolix’s effect on EHP-30 self-image domain

**Figure 11:**
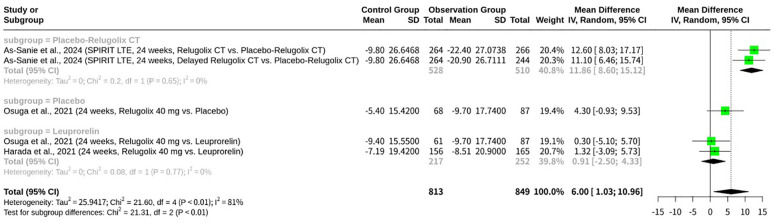
Forest plot of odds ratio of relugolix’s effect on EHP-30 self-image domain.

This meta-analysis, shown as **Figure 11**, investigated the impact of Relugolix on EHP-30 Self-image domain scores, pooling data from five datasets. The overall REM revealed a significant positive mean difference (MD = 6.00, 95%-CI: 1.03; 10.96, p=0.0179), suggesting an improvement in self-image with Relugolix treatment. However, substantial heterogeneity was observed across the included studies (I^2^ = 81.5%, p=0.0002). A subgroup analysis, categorized by the control group’s intervention, demonstrated a highly significant difference between subgroups (p<0.0001). Specifically, Relugolix showed a significant improvement in self-image scores when compared to placebo or delayed Relugolix combination therapy (MD = 11.86, 95%-CI: 8.60; 15.12 for the “Placebo-Relugolix CT” subgroup, I^2^ = 0.0%; MD = 4.30, 95%-CI: -0.93; 9.53 for the “Placebo” subgroup), with no heterogeneity within these subgroups. Conversely, when compared against Leuprorelin, another active GnRH agonist, Relugolix had no significant effect on self-image (MD = 0.91, 95%-CI: -2.50; 4.33), with the confidence interval crossing zero.

Self-image, defined as an individual’s mental picture of their abilities, appearance, and personality, is a key component of psychological well-being and is closely related to self-esteem (11). In clinical trials, the Self-image domain is a vital metric for assessing how treatments impact a patient’s self-perception (11). For example, the study examined improvements in the self-image domain over 104 weeks for Relugolix, likely comparing the treatment group to a placebo (11). The data from such studies can show notable changes, with one instance mentioning a score change of 46 points, which could signify a significant shift in self-perception due to the intervention (11). The literature also suggests that other numbers, such as “15,” might refer to a specific item on a questionnaire or a score on an assessment tool, indicating a need for more context to interpret fully (12). These findings underscore the importance of the EHP-30 Self-image domain as a measure of a therapy’s comprehensive impact on a patient’s mental and emotional state.

### Adverse events and safety profile

The safety profiles of oral GnRH antagonists were consistently evaluated across the included studies. Treatment-emergent adverse events (TEAEs) were the primary safety endpoint, with most studies employing the Medical Dictionary for Regulatory Activities (MedDRA) for coding and the National Cancer Institute’s Common Terminology Criteria for Adverse Events (CTCAE) for grading severity (12). The most common adverse events reported were primarily linked to the drugs’ hypoestrogenic effects. Hot flushes were a frequently reported major adverse event with relugolix, and their prevalence was found to be dose-dependent (13). Other common TEAEs included headache, fatigue, musculoskeletal pain, and nasopharyngitis (12–14). A comparison between relugolix and the GnRH agonist leuprorelin in a phase 3 study for endometriosis-associated pain showed comparable safety profiles, with both drugs causing similar rates of hot flushes, metrorrhagia, and headache (15). However, relugolix, being a GnRH antagonist, avoided the initial “flare-up” of symptoms that is characteristic of GnRH agonists.

Discontinuations due to adverse events were generally low. In a pivotal clinical trial, two patients discontinued treatment due to TEAEs (15), while in the SPIRIT 1 and SPIRIT 2 trials, the number of discontinuations ranged from 7 to 11 patients in the relugolix combination groups (12). Serious adverse events were rare, with one study reporting five serious AEs and no fatal events across all groups (12). Long-term safety assessments, particularly in the long-term extension (LTE) studies of relugolix combination therapy, also focused on bone mineral density (BMD) changes. As expected, a dose-dependent decrease in BMD was observed with relugolix, similar to that of leuprorelin, due to the induced hypoestrogenic state (13) (14). These findings highlight the importance of add-back therapy in managing the long-term hypoestrogenic effects while maintaining therapeutic efficacy.

## Discussion

The observed efficacy of GnRH antagonists in alleviating EAP stems from their direct and rapid mechanism of action. Unlike GnRH agonists, which initially cause a ‘flare-up’ effect before downregulating receptors, antagonists induce an immediate, dose-dependent blockade of GnRH receptors in the pituitary (16, 17). This direct inhibition rapidly suppresses the pulsatile release of luteinizing hormone and follicle-stimulating hormone, leading to a significant reduction in ovarian estrogen and progesterone production (1). Endometriosis is a predominantly estrogen-dependent condition, where the presence of endometrial-like tissue outside the uterus is sustained and stimulated by ovarian hormones (18, 19). By effectively reducing estrogen levels, GnRH antagonists induce atrophy of these ectopic lesions, thereby diminishing inflammation, lesion growth, and nerve sensitization, which are key contributors to EAP, including dysmenorrhea, non-menstrual pelvic pain, and dyspareunia (18, 20).

Crucially, the development of oral, non-peptide GnRH antagonists, such as elagolix, relugolix, and linzagolix, has marked a significant advancement, offering improved convenience and patient adherence compared to parenteral administration (17). Furthermore, the ability to tailor estrogen suppression through varying doses allows for personalized treatment, balancing pain relief with the mitigation of hypoestrogenic side effects. The integration of add-back therapy (ABT), typically involving low-dose estrogen and progestin, alongside higher doses of GnRH antagonists, represents a strategic approach to overcome the long-term limitations associated with profound estrogen deprivation, such as bone mineral density loss and vasomotor symptoms, without compromising the therapeutic efficacy in pain reduction (21). This combination therapy has been shown to sustain pain relief while minimizing adverse effects, enabling extended treatment durations as evidenced by long-term extension studies (22). Therefore, the mechanism of GnRH antagonists, particularly when combined with ABT, offers a powerful and adaptable strategy for managing the complex pain symptoms of endometriosis.

The meta-analysis reveals significant overall heterogeneity (I^2^ = 90.7%), highlighting a considerable variation in treatment effects across the included studies. The subgroup analysis, meticulously stratified by the type of comparator arm, offers crucial insights into the sources of this heterogeneity, demonstrating that the choice of control group significantly influences the observed efficacy of Relugolix in managing EAP, as measured by EHP-30 pain domain scores. The “Placebo-Relugolix CT” subgroup, which includes the SPIRIT 1 and SPIRIT 2 pivotal trials (Giudice et al., 2022) and their extension (As-Sanie et al., 2024), exhibits a high degree of within-subgroup heterogeneity (I^2^ = 89.1%). These studies largely evaluate Relugolix combination therapy (Relugolix, estradiol, and norethisterone acetate) against a placebo or a delayed combination therapy, designed to mitigate hypoestrogenic side effects. The large mean difference (MD = 8.86) in favor of Relugolix combination therapy in this subgroup underscores its substantial clinical benefit compared to placebo, as expected from effective treatments. In contrast, the “Placebo” subgroup, primarily comprising the Osuga et al., 2020 and 2021 studies, which investigated Relugolix monotherapy against a simple placebo control, shows a notably larger mean difference (MD = 15.31) and reduced, albeit still moderate, heterogeneity (I^2^ = 41.6%). The greater effect size here is likely attributable to the direct comparison with a pure placebo, without the add-back hormonal component in the experimental arm, leading to a more pronounced difference in pain reduction. Finally, the “Leuprorelin” subgroup, encompassing comparisons of Relugolix monotherapy against an active comparator, Leuprorelin (Osuga et al., 2020, 2021; Harada et al., 2021), exhibits the lowest heterogeneity (I^2^ = 22.8%) and a negative mean difference (MD = -3.79). This indicates that Relugolix monotherapy may be marginally less effective than Leuprorelin in reducing EHP-30 pain scores. The reduced heterogeneity within this subgroup is particularly insightful. Both Relugolix and Leuprorelin are GnRH analogs, and while their mechanisms of action differ (antagonist vs. agonist), they ultimately achieve similar therapeutic effects by suppressing ovarian hormone production. The head-to-head comparison between two active treatments with similar pharmacological goals could naturally lead to more consistent, and thus less heterogeneous, results. The “double-dummy” design employed in the Harada et al., 2021 and Osuga et al., 2020 studies, where both active and placebo versions of oral and injectable drugs were used, ensured robust blinding and comparability between these two active treatment arms. The finding that Relugolix performs slightly worse than Leuprorelin in this subgroup, while having low heterogeneity, further strengthens the conclusion that the choice of comparator profoundly shapes the perceived efficacy and the overall landscape of a meta-analysis. This analysis underscores the critical importance of control group selection in interpreting the aggregated treatment effects and understanding sources of variability in meta-analytic findings.

The finding that Relugolix showed no significant difference in effect compared to Leuprorelin across multiple EHP-30 domains (Pain, Control and Powerlessness, Emotional Well-being, and Social Support) is a crucial observation. This result, while not reaching statistical significance in favor of Relugolix, could be interpreted in two ways. First, it may suggest therapeutic equivalence between these two GnRH-modulating therapies. Leuprorelin, a well-established GnRH agonist, has long been a standard of care for endometriosis-associated pain. The lack of a significant difference implies that Relugolix, an oral GnRH antagonist, may offer a comparable level of clinical benefit without the initial “flare-up” effect characteristic of agonists. This would present a valuable alternative for clinicians and patients seeking effective symptom control. However, it is also essential to consider the influence of methodological factors. The number of studies in the Leuprorelin subgroup was very limited, which may have reduced the statistical power to detect a true difference between the two drugs. Furthermore, while the included studies were RCTs, some design elements could have masked potential distinctions. For example, variations in blinding methods (some studies were open-label extension trials) and differences in patient populations (e.g., studies conducted exclusively in Japan vs. multinational trials) could have introduced confounding variables. These factors, alongside the inherent differences in the mechanism of action (the initial agonistic flare-up with Leuprorelin versus the direct antagonism with Relugolix) suggest that a nuanced interpretation is needed. The meta-analysis results, therefore, do not definitively establish therapeutic equivalence, but rather highlight the need for further, more direct comparative trials with larger sample sizes to conclusively determine if one drug offers a superior long-term advantage over the other.

Intriguingly, the subgroup analysis, which categorized studies by the experimental Relugolix dosage (10 mg, 20 mg, and 40 mg), did not reveal a significant difference in treatment outcomes among these dose groups (Q = 1.37, d.f. = 2, p=0.5031). While the 40 mg subgroup exhibited a numerically larger mean difference in pain reduction (MD = 8.49; 95%-CI: 4.54; 12.44) compared to the 10 mg (MD = 2.55; 95%-CI: -8.52; 13.63) and 20 mg (MD = 4.09; 95%-CI: -6.91; 15.09) subgroups, this apparent trend did not translate into a distinct effect. This finding suggests that, within the tested range, the dose of Relugolix may not be a primary determinant of the magnitude of pain relief in endometriosis. Several factors could contribute to this observed lack of statistical differentiation among dosages. Firstly, the study designs referenced (e.g., Osuga et al., 2020; Osuga et al., 2021) often included multiple Relugolix dosages within the same trial, allowing for direct comparisons. However, the sample sizes within each subgroup might still be insufficient to detect more subtle, yet clinically relevant, dose-dependent differences, particularly given the high overall heterogeneity. Secondly, the pain experience in endometriosis is multifactorial and can be influenced by various individual patient characteristics and disease severities, as evidenced by the broad inclusion criteria in studies like Harada et al., 2021, which allowed for diverse patient populations with varying degrees of dysmenorrhea, pelvic pain, and diagnostic confirmations (laparotomy/laparoscopy confirmed, ovarian endometrioma, or clinical endometriosis). The inherent variability in baseline pain perception and response to treatment among such diverse patient cohorts could mask potential dose-response relationships. Furthermore, the mechanism of action of GnRH antagonists like Relugolix primarily involves suppression of estradiol to therapeutic levels, thereby alleviating EAP. It is plausible that even the lower doses of Relugolix achieve sufficient hormonal suppression to exert a significant therapeutic effect on pain. Once a certain threshold of estrogen suppression is met, further increases in dosage might not yield proportionately greater pain reduction, especially if pain pathways involve other factors beyond hormonal influences. Future research, perhaps with larger, more targeted dose-ranging studies or investigations into patient-specific biomarkers, could help elucidate if specific patient subgroups benefit from higher doses or if a ceiling effect for pain relief is reached at lower therapeutic doses of Relugolix.

While our subgroup analysis indicated no significant difference in EHP-30 pain domain scores between Relugolix combination therapy and Relugolix monotherapy (p=0.2740), it is crucial to discuss the numerical trends and potential underlying reasons for this observation. The combination therapy subgroup consistently showed a numerically greater mean difference in pain reduction (MD = 8.86) compared to monotherapy (MD = 4.99). Furthermore, the 95% confidence interval for Relugolix monotherapy crossed zero ([-0.78; 10.77]), suggesting a less conclusive individual effect for monotherapy in pain reduction compared to the more robust and clearly positive effect observed with combination therapy. This numerical difference, even if not significant in this meta-analysis, aligns with the clinical rationale behind developing combination therapies. Relugolix, as a GnRH antagonist, potently suppresses ovarian estrogen production, which is key to its efficacy in EAP relief. However, this profound estrogen suppression can lead to hypoestrogenic side effects, including hot flashes and, critically, bone mineral density (BMD) loss, as observed in studies like Osuga et al., 2020, and Osuga et al., 2021. Combination therapy, incorporating low-dose estradiol and norethisterone acetate (as seen in Giudice et al., 2022, and As-Sanie et al., 2024), is specifically designed as an “add-back” regimen. The primary aim of this add-back is not to enhance direct pain suppression but to mitigate these dose-dependent hypoestrogenic side effects, thereby improving tolerability and enabling longer-term treatment, which is often necessary for chronic conditions like endometriosis. The less conclusive effect of monotherapy, reflected by its confidence interval spanning zero, could stem from several factors. Patients on monotherapy might experience more pronounced hypoestrogenic symptoms, leading to poorer adherence or higher dropout rates, which could, in turn, attenuate the observed pain relief in the long run. The *SPIRIT* trials (Giudice et al., 2022; As-Sanie et al., 2024), for instance, explicitly incorporated combination therapy from the outset or via a delayed combination arm, recognizing the need to balance efficacy with long-term safety and tolerability. While the direct pain-modulating effect of Relugolix itself is likely similar across doses and formulations once effective estrogen suppression is achieved, the overall patient experience and sustained adherence, which are crucial for consistent pain management, could be superior with combination therapy due to its favorable safety profile. Therefore, although statistical significance for direct pain efficacy differences between the two approaches was not reached in our analysis, the clinical benefit of combination therapy in terms of sustained patient compliance and overall quality of life might explain the numerically more robust pain reduction observed with CT and its clearer statistical significance.

The stratified meta-analysis by follow-up duration provides initial insights into the long-term efficacy of Relugolix, yet the question of whether the drug maintains a sustained pain-inhibiting effect over prolonged periods remains challenging to definitively answer from the current meta-analysis. While the 24-week follow-up subgroup showed a clear and significant mean difference in pain reduction, the data for longer follow-up periods (52-week and 104-week) present a less conclusive picture. For both the 52-week and 104-week subgroups, the respective mean differences were numerically smaller (MD = 1.81 and MD = 2.41) compared to the 24-week group. Crucially, these long-term findings are based on only two studies per subgroup, resulting in low statistical power, and their respective 95% confidence intervals were wide and both crossed zero (52-week: [-0.81; 4.43]; 104-week: [-0.17; 4.99]). This indicates that a significant benefit in EHP-30 pain scores could not be conclusively demonstrated at these extended durations. Therefore, it is important to emphasize this limitation and avoid overgeneralizing the long-term effects of Relugolix based on the current evidence. A primary limitation in interpreting these longer-term results is the considerably reduced number of studies available for analysis, with only two observations each for the 52-week and 104-week subgroups. This limited sample size drastically reduces the statistical power to detect smaller, but potentially clinically meaningful, effects and makes any conclusions regarding true heterogeneity (I^2^ = 0.0%) unreliable. The absence of detected heterogeneity in these small subgroups is more likely an artifact of insufficient data rather than a genuine indication of uniform treatment effects. The observation that the overall test for subgroup differences across all follow-up durations did not reach statistical significance (p=0.1104) technically implies no significant variation in effect over time based on the available data. However, the numerical attenuation of the mean difference and the loss of statistical significance in the longer-term subgroups hint at a potential decrease in the magnitude or consistency of effect, or perhaps an increased variability in response that cannot be captured with limited studies. For a chronic condition like endometriosis, the persistence of pain relief is paramount. Although Relugolix has demonstrated significant short-to-medium term efficacy, the current meta-analysis highlights the critical need for more robust, large-scale, and sufficiently powered long-term randomized controlled trials (beyond 24 weeks) to ascertain the durable efficacy of Relugolix in pain inhibition. Such studies would be essential to determine if the initial benefits are sustained, to characterize the true long-term variability in patient response, and to better inform clinical guidelines for extended Relugolix use in managing EAP.

The meta-analyses consistently reveal Relugolix’s multifaceted impact on the HRQoL for women with endometriosis, as assessed by various EHP-30 domains. Across the EHP-30 Control and Powerlessness, Emotional Well-being, Social Support, and Self-image domains, Relugolix demonstrated significant improvements when compared to placebo or delayed Relugolix combination therapy, with the effects in these placebo-controlled subgroups often showing low internal heterogeneity. This robust efficacy against non-active comparators underscores Relugolix’s ability to positively influence patients’ perceived control over their condition, emotional state, social interactions, and self-perception. However, a critical insight emerged from the subgroup analyses involving Leuprorelin: in all four EHP-30 domains (Emotional Well-being, Control and Powerlessness, Self-image, Social Support), Relugolix did not show a significant superiority when compared to this active GnRH agonist. While some numerical trends were observed, the confidence intervals consistently crossed zero, indicating that Relugolix’s benefits on these specific quality-of-life aspects are comparable to, or not significantly better than, those achieved by Leuprorelin. The high overall heterogeneity noted in these meta-analyses is thus largely attributable to the distinct effects observed when comparing Relugolix against an inert control versus another active GnRH agonist.

The finding that the 52-week and 104-week follow-up subgroups did not show a statistically significant effect on EHP-30 pain scores, while also having very limited data points, requires careful interpretation for clinical practice. Clinicians should be cautious about extrapolating the robust short-term benefits of Relugolix to long-term pain management. The wide confidence intervals that cross zero for these longer durations do not necessarily indicate a lack of efficacy, but rather an inconclusive finding due to the small sample size. Given the high heterogeneity in shorter-term data, it’s plausible that a true long-term effect exists but is masked by the insufficient number of studies. Therefore, for clinicians, this meta-analysis suggests that while Relugolix is highly effective for reducing pain in the short to medium term (up to 24 weeks), its long-term efficacy beyond one year cannot be definitively established from the current evidence base. Patient selection should be a key consideration. For individuals requiring extended treatment, clinicians should weigh the known short-term benefits against the uncertain long-term outcomes and potential risks of prolonged use. This highlights a critical gap in the existing literature, underscoring the need for more long-term, well-designed randomized controlled trials to better understand the sustained efficacy and safety of Relugolix for chronic endometriosis-associated pain. Until such data become available, clinical decisions on extended use should be individualized, closely monitoring patient-reported outcomes while considering alternative or adjunctive therapies as needed.

Despite providing valuable insights into Relugolix’s efficacy, this meta-analysis is subject to several limitations. Firstly, substantial heterogeneity (I^2^ values frequently above 70-90%) was observed in several overall analyses for EHP-30 domains, suggesting significant unmeasured differences between studies. While subgrouping by comparator type explained a portion of this variability, residual heterogeneity or limitations in assessing heterogeneity in very small subgroups persist. Secondly, the number of studies available for certain subgroups, particularly those with longer follow-up durations (52-week and 104-week) and direct comparisons against active comparators like Leuprorelin, was considerably limited. This scarcity of data restricts the statistical power to draw definitive conclusions regarding long-term sustained efficacy or precise comparative effectiveness against other established treatments. Thirdly, our risk of bias assessment indicated an unclear or high risk of bias for key study design elements such as blinding and selective reporting. The absence of individual patient data further limited our ability to assess baseline comparability across studies or to adjust for potential confounding variables. Therefore, it is important to acknowledge that our findings may be susceptible to selection and reporting bias, and we underscore the critical need for future meta-analyses based on individual patient data to provide more precise and robust estimates of treatment effects.

## Conclusion

In conclusion, this meta-analysis provides robust evidence for the efficacy of Relugolix in managing EAP and improving various aspects of health-related quality of life. Relugolix consistently demonstrated a significant reduction in EHP-30 pain domain scores and an increased proportion of EHP-30 pain responders when compared to placebo. Furthermore, significant improvements were observed across other EHP-30 quality-of-life subdomains, including Control and Powerlessness, Emotional Well-being, Social Support, and Self-image, when Relugolix was compared against placebo or delayed combination therapy. While numerically Relugolix combination therapy tended to show a greater effect on pain reduction than monotherapy, this difference was not significant. Importantly, when compared against Leuprorelin, another active GnRH agonist, Relugolix’s efficacy in improving these EHP-30 quality-of-life aspects was generally comparable and not statistically superior. Overall, these findings suggest that Relugolix offers significant symptomatic and quality-of-life benefits for women with endometriosis, largely driven by its efficacy against inactive comparators, providing a therapeutic profile similar to other effective GnRH agonists. Future research should focus on longer-term trials and direct comparative effectiveness studies to solidify its position among existing treatments.

## Data Availability

The original contributions presented in the study are included in the article/supplementary material. Further inquiries can be directed to the corresponding author.

## References

[1] Othman Essam R Al-Hendy A Mostafa R Lambalk Cornelis B Mijatovic V . Oral gnRH antagonists in combination with estradiol and norethindrone acetate for pain relief associated with endometriosis: A review of evidence of a novel class of hormonal agents. Int J women’s Health. (2024) 16:309–21. doi: 10.2147/IJWH.S442357, PMID: 38435758 PMC10908275

[2] Wu D Hu M Hong Li Hong S Ding W Min J . Clinical efficacy of add-back therapy in treatment of endometriosis: a meta-analysis. Arch gynecology obstetrics. (2014) 290:513–23. doi: 10.1007/s00404-014-3230-8, PMID: 24728145

[3] Jeng C-J Chuang L Shen J . A comparison of progestogens or oral contraceptives and gonadotropin-releasing hormone agonists for the treatment of endometriosis: a systematic review. Expert Opin pharmacotherapy. (2014) 15:767–73. doi: 10.1517/14656566.2014.888414, PMID: 24588662

[4] Xin L Ma Y Ye M Chen L Liu F Hou Q . Efficacy and safety of oral gonadotropin-releasing hormone antagonists in moderate-to-severe endometriosis-associated pain: a systematic review and network meta-analysis. Arch gynecology obstetrics. (2023) 308:1047–56. doi: 10.1007/s00404-022-06862-0, PMID: 36656435 PMC10435625

[5] Yan H Shi J Li X Dai Y Wu Y Zhang J . Oral gonadotropin-releasing hormone antagonists for treating endometriosis-associated pain: a systematic review and network meta-analysis. Fertility sterility. (2022) 118:1102–16. doi: 10.1016/j.fertnstert.2022.08.856, PMID: 36283862

[6] Nogueira Neto J Melo VG Lima LCS Costa MVLR Silva LC Gomes LMRS . Improved quality of life (EHP-30) in patients with endometriosis after surgical treatment. Rev da Associacao Med Bras. (1992) . 2023 69:e20230316. doi: 10.1590/1806-9282.20230316, PMID: 37585993 PMC10427168

[7] Bafort C Beebeejaun Y Tomassetti C Bosteels J Duffy JM . Laparoscopic surgery for endometriosis. Cochrane Database systematic Rev. (2020) 10:CD011031. doi: 10.1002/14651858.CD011031.pub3, PMID: 33095458 PMC8428328

[8] Moher D Liberati A Tetzlaff J Altman Douglas G . Preferred reporting items for systematic reviews and meta-analyses: the PRISMA statement. Ann Internal Med. (2009) 151:264–9, W64. doi: 10.7326/0003-4819-151-4-200908180-00135, PMID: 19622511

[9] Hooijmans Carlijn R Rovers Maroeska M de Vries Rob BM Leenaars M Ritskes-Hoitinga M Langendam Miranda W . SYRCLE’s risk of bias tool for animal studies. BMC Med Res Method. (2014) 14:43. doi: 10.1186/1471-2288-14-43, PMID: 24667063 PMC4230647

[10] Balduzzi S Rücker G Schwarzer G . How to perform a meta-analysis with R: a practical tutorial. Evidence-Based Ment Health. (2019) 22:153–60. doi: 10.1136/ebmental-2019-300117, PMID: 31563865 PMC10231495

[11] As-Sanie S Abrao Mauricio S Reznichenko G Wilk K Zhong Y Perry J . Impact of relugolix combination therapy on functioning and quality of life in women with endometriosis-associated pain. Fertility sterility. (2024) 122:687–95. doi: 10.1016/j.fertnstert.2024.06.009, PMID: 38906210

[12] Giudice Linda C As-Sanie S Arjona Ferreira Juan C Becker Christian M Abrao Mauricio S Lessey Bruce A . Once daily oral relugolix combination therapy versus placebo in patients with endometriosis-associated pain: two replicate phase 3, randomised, double-blind, studies (SPIRIT 1 and 2). Lancet (London England). (2022) 399:2267–79. doi: 10.1016/S0140-6736(22)00622-5, PMID: 35717987

[13] Osuga Y Seki Y Tanimoto M Kusumoto T Kudou K Terakawa N . Relugolix, an oral gonadotropin-releasing hormone receptor antagonist, reduces endometriosis-associated pain in a dose-response manner: a randomized, double-blind, placebo-controlled study. Fertility sterility. (2021) 115:397–405. doi: 10.1016/j.fertnstert.2020.07.055, PMID: 32912633

[14] Osuga Y Seki Y Tanimoto M Kusumoto T Kudou K Terakawa N . Relugolix, an oral gonadotropin-releasing hormone (GnRH) receptor antagonist, in women with endometriosis-associated pain: phase 2 safety and efficacy 24-week results. BMC women’s Health. (2021) 21:250. doi: 10.1186/s12905-021-01393-3, PMID: 34154590 PMC8218467

[15] Harada T Osuga Y Suzuki Y Fujisawa M Fukui M Kitawaki J . Relugolix, an oral gonadotropin-releasing hormone receptor antagonist, reduces endometriosis-associated pain compared with leuprorelin in Japanese women: a phase 3, randomized, double-blind, noninferiority study. Fertility sterility. (2022) 117:583–92. doi: 10.1016/j.fertnstert.2021.11.013, PMID: 34895700

[16] Coccia ME Comparetto C Bracco GL Scarselli G . GnRH antagonists. Eur J obstetrics gynecology Reprod Biol. (2004) 115 Suppl 1:S44–56. doi: 10.1016/j.ejogrb.2004.01.033, PMID: 15196716

[17] Padula AM . GnRH analogues–agonists and antagonists. Anim Reprod Sci. (2005) 88:115–26. doi: 10.1016/j.anireprosci.2005.05.005, PMID: 15955640

[18] Taylor Hugh S Giudice Linda C Lessey Bruce A Abrao Mauricio S Kotarski J Archer David F . Treatment of endometriosis-associated pain with elagolix, an oral gnRH antagonist. New Engl J Med. (2017) 377:28–40. doi: 10.1056/NEJMoa1700089, PMID: 28525302

[19] Taylor Hugh S Kotlyar Alexander M Flores Valerie A . Endometriosis is a chronic systemic disease: clinical challenges and novel innovations. Lancet (London England). (2021) 397:839–52. doi: 10.1016/S0140-6736(21)00389-5, PMID: 33640070

[20] Alonso A Gunther K Maheux-Lacroix S Abbott J . Medical management of endometriosis. Curr Opin obstetrics gynecology. (2024) 36:353–61. doi: 10.1097/GCO.0000000000000983, PMID: 39159261 PMC11356688

[21] Viviano M Benagiano G Guo S-W Pluchino N . Why do oestrogens matter: systematic review and meta-analysis assessing GnRH antagonists, considering add-back therapy, for endometriosis-associated pain. Reprod biomedicine. (2024) 49:104321. doi: 10.1016/j.rbmo.2024.104321, PMID: 39098266

[22] Blair Hannah A . Relugolix/estradiol/norethisterone acetate: A review in endometriosis-associated pain. Drugs. (2024) 84:449–57. doi: 10.1007/s40265-024-02018-3, PMID: 38592603 PMC11127801

